# Reduction in Fatigue Symptoms Following the Administration of Nutritional Supplements in Patients with Multiple Sclerosis

**DOI:** 10.3390/medsci9030052

**Published:** 2021-07-20

**Authors:** Pasquale Ferorelli, Francesco Antonelli, Anna Shevchenko, Carlo Mischiati, Manfred Doepp, Stefano Lenzi, Ilaria Borromeo, Giordana Feriotto, Simone Beninati

**Affiliations:** 1 Scientific Research Institute “CRSC”, 20100 Milan, Italy; CRSCresearch@hotmail.com; 2 Scientific Association “ARSS”, 00100 Rome, Italy; francescoantonelli39@yahoo.com; 3 Department of Pharmacology, Kabardine University, 101000 Nalchik, Russia; kabardineuniversity@yahoo.com; 4 Department of Neuroscience and Rehabilitation, University of Ferrara, 44100 Ferrara, Italy; msc@unife.it; 5 Department of Psychology and Sports Science, Giessen Justus, Liebig University Gießen, 35398 Gießen, Germany; holisticcenter1@yahoo.de; 6 Department of Health Engineering, Université Européenne de Bruxelles Jean Monnet, Schaerbeek, 1030 Brussels, Belgium; stefano.lenzi@yahoo.it; 7 Department of Physics, University of Tor Vergata, 00100 Rome, Italy; ilaria18scv@hotmail.it; 8 Department of Chemical, Pharmaceutical and Agricultural Sciences, University of Ferrara, 44100 Ferrara, Italy; frn@unife.it; 9 Department of Biology, University of Rome Tor Vergata, Via della Ricerca Scientifica, 00100 Rome, Italy

**Keywords:** multiple sclerosis, fatigue, dietary supplements, vitamins, QOL

## Abstract

Despite recent advances in immune-modulatory drugs, pharmacological therapies have been proven ineffective in severe presentations of multiple sclerosis (MS), including secondary progressive MS. At present, therapeutic interventions’ performance is primarily focused on ameliorating symptoms to improve the patient’s quality of life (QOL). Among complementary treatments, nutrition has been considered a decisive factor to control symptoms and enhance the wellness of MS patients. Although no special diets are associated with MS, the impact of diet and dietary supplements on the course of progressive forms of the disease has been studied during the last few years. Fatigue is among the most common and disabling symptoms reported by MS patients. Fatigue has been defined in the Multiple Sclerosis Council for Clinical Practice Guidelines (MSCCPG, 1998) as a “subjective lack of physical and/or mental energy that the individual perceives as an interference with habitual and desired activities”. This study aimed to compare the psychometric functioning of the “Fatigue Severity Scale” (FSS) and the “Modified Fatigue Impact Scale” (MFIS) in our sample of people with MS. Specifically, during chronic treatment, the change in these two parameters with two vitamin-rich dietary supplements (Citozym^®^ and Ergozym^®^) was evaluated. The impact of these nutritional supplements revealed differences in antioxidant and anti-inflammatory parameters among the volunteers in the treatment group, with a subsequent improvement in fatigue. In conclusion, the results obtained have confirmed the effectiveness of complementary nutritional therapies, evaluated essentially based on hematological biomarkers, through which it is possible to act on disability to improve the QOL of MS patients.

## 1. Introduction

Multiple sclerosis (MS) and acute disseminated encephalitis, acute necrotic hemorrhagic encephalitis, and myelinopathy represent a set of diseases characterized by demyelination areas in the central nervous system, with an inflammatory response and a consequent neurological course of great importance, leading to severe disability [1]. Modifiable factors, including sunlight exposure (vitamin D), obesity at a young age, and dietary intake, have been associated with the pathogenesis of the disease process [2].

In normal aerobic cells, oxidative damage is prevented by an efficient antioxidant defense. Insufficient antioxidant protection or the excessive production of reactive oxygen species (ROS) generates a condition known as oxidative stress (OS), which can lead to cellular damage through the action of oxidizing cellular components, including lipids, proteins, and DNA. Myelin destruction plays the primary role in disability in MS patients, and myelin sheaths are highly sensitive to OS [3,4]. 

Currently, no effective clinical indications for applying dietary supplements as a complementary treatment against MS symptoms are available. Examining the therapeutic effects of a range of nutritional supplements suitable for controlling cell metabolism in relapsing and progressive MS forms can be an interesting approach to complement the limited range of treatments available [5]. Similarly, the impact of dietary intervention on inflammation can be enhanced by supplements with intense antioxidant activities. Despite the current therapies aimed at improving the disease, poor QOL in MS patients remains a significant problem, and fatigue is one of the common and disabling symptoms [6]. Patients with MS have a significantly reduced QOL compared with the general population. Some studies have reported that a reduced QOL may be partly the consequence of neurological disability. Fatigue represents the most frequent and disabling symptom among patients with MS, markedly interfering with daily life. Primary fatigue’s pathophysiology remains unknown, but undoubtedly, inflammation-related OS and immunologic factors play a central role. The treatment of this symptom to improve the QOL presently remains a challenge. 

Disability, depression, and fatigue are independent predictors of life quality in people with MS [6]. Fatigue is one of the most common symptoms of multiple sclerosis and may even be the first sign of the disease. Fatigue affects between 75 and 95% of patients; it is not strictly correlated with gender, race, and education. It is considered by 55% of patients as one of the most disabling symptoms [7]. However, its diagnosis is not easy because it represents a subjective state, difficult to describe and be understood by others. It interferes with a person’s physical and mental activities with MS and contributes to the worsening of the difficulties already present, negatively affecting life quality. Fatigue can have a psychological impact on a person, especially when fatigue and lack of strength make it harder to perform even the simplest tasks.

Effective treatment options for fatigue remain limited. In this observational study it was noted that, among the multiple symptoms of volunteers with MS, fatigue was one of the most painful complaints that patients may experience throughout their lives. The pathogenesis of fatigue and its primary causes remains obscure, and there are currently no adequate therapies. One of the main obstacles to understanding this symptom was the absence of a universally accepted measurement method that could quantify an experience, often disabling and elusive, properly. Without a measurement, progress in fatigue therapy remains limited. Currently, this limitation has been overcome by employing two assessment methods [8], such as the psychometric functioning of “Fatigue Severity Scale” (FSS) and the “Modified Fatigue Impact Scale” (MFIS), which is part of the Multiple Inventory QOL (MSQLI), a battery consisting of 10 individual scales providing a QOL measure that is both generic and MS-specific [9]. The MFIS is a modified version of the 40-item FSS, which was originally developed to assess the effects of fatigue on the cognitive and psychosocial aspects, related to the QOL, in patients with chronic diseases, specifically MS.

Several factors could contribute to fatigue development and/or exacerbation, and this requires a careful work-up to search for all the possible underlying causes, such as sleep disorders, endocrine dysfunction, and mood disorders, to name a few. Central fatigue is described as fatigue not from the muscle itself but rather from the central nervous system (CNS) and the transmission of signals from the brain to the muscle [10]. Therefore, central fatigue is related to the brain and spinal cord.

Nutrition is considered a possible factor in the pathogenesis of MS. Current studies in nutritional interventions suggest that diet may be regarded as a complementary treatment to control disease progression [11]. Although several observational studies have demonstrated a relationship between specific dietary patterns and the prevalence of MS, very few have shown a correlation of the diet with fatigue and QOL, and even fewer have correlated nutritional intake with biological markers in people with MS. Low levels of specific micronutrients, including vitamins D, B12, and A, have also been shown to contribute to the pathogenesis of MS, and found that diets lower in folate and magnesium correlate to increased fatigue in MS [5]. The purpose of this observational study was to relate the intake of two nutritional supplements containing several vitamins and folic acid, among other numerous micro-nutrients, to fatigue in patients with MS.

## 2. Materials and Methods

### 2.1. Study Design

This observational study was conducted on 50 patients with MS to determine the effects on the QOL following the administration of two commercial dietary supplements, called Citozym^®^ and Ergozym^®^, (Citozeatec Italia-FDA registration 12932524008 Pin no. bfJ3h263). The study was conducted according to the guidelines of the Declaration of Helsinki, and was reviewed and approved on the July 20th, 2020, by the board of the “CRSC” Research Institute, under protocol No. “AB2745P28” as part of the “Dietary Supplementation Project”.

The two nutritional supplements contain several vitamins: Citozym^®^: vitamins/100 g of product: C (ascorbic acid) 550 mg; B5 (pantotenic acid) 56 mg; D (calciferol) 56 µg; B9 (folic acid) 3.3 mg. Ergozym^®^: vitamins/100 g of product: A, 9.3 mg; B3/PP, 100 mg; B2 (riboflavin) 25 mg; B6 (Pyridoxine) 33 mg; B8/H (biotin) 200 mg; B9 (folic acid) 2 mg; B12 (cianocobalamine) 25 µg). Fifty female volunteers with a “Kurtzke Extended disability status score” (EDSS) of less than 6 were recruited for the study, with a definitive diagnosis of the relapsing–remitting form of MS. The EDSS is widely used to measure and assess the clinical characteristics of multiple sclerosis patients. It is also a widely accepted tool in clinical trials, for example, to assess the effect of treatments on disease progression [12]. The total EDSS score is determined by the following two factors: walking ability and scores for eight functional systems. A subscale that assesses the functional status of certain functional systems that are variably affected by the disease is used. A total of 25 volunteers received the two dietary supplements for 70 days, according to an experimental nutritional protocol, and 25 received a placebo preparation made with distilled water, honey, and permitted food pigments, with the same color as that of Citozym^®^ and Ergozym^®^ and were considered as a negative control. Written informed consent was obtained from all study participants. Patients with secondary or primary progressive MS, pregnancy, corticosteroid treatment, or who simultaneously suffered from another chronic disease such as rheumatic disease, severe heart disease, malignant cancers, and other neurologic and inflammatory diseases were excluded. Patients were advised not to discontinue their routine medications. A consent report form was completed before the study for all patients. After the patient’s objective examination and history, serum blood tests were prescribed to check the inflammatory and oxidative statuses to exclude the presence of diseases whose symptoms might overlap with those of MS. The participants were then randomized into two dietary supplementation groups for age, height, weight, BMI, and duration of MS (difference between groups, *p* < 0.01). Demographic and anthropometric characteristics expressed as mean and standard deviation were collected from the patients’ medical history. To ascertain the baseline values of FSS and MFIS, the two groups of volunteers (placebo-treated and supplement-treated) were subjected to the two psychometric tests at the beginning of the treatment. Fatigue symptoms were quantified using the psychometric tests FSS and MFIS in both groups, at day 0, 5, 10, 20, 35, 50, 60, and 70 from the start of supplementation. FSS consisted of 9 questions, each with a score from 1 to 7, where 7 was the worst situation (total score range: 9–63). MIFS consisted of 21 questions, each scoring from 0 to 4, with 4 being the worst situation (total score range: 0–84). Items on the MFIS can be aggregated into three subscales (physical, cognitive, and psychosocial) and a total MFIS score. All items are scaled to ensure that higher scores indicate a more significant impact of fatigue on a person’s activities. In this experimental trial, only the physical and total MFIS scores were considered [13].

No specific blood tests are available for the diagnosis of MS. However, after the patient’s objective examination and history, a series of blood tests were prescribed with a view to a differential diagnosis, the purpose of which was not to confirm the presence of MS, but to check the inflammatory status and OS levels to preclude the presence of diseases whose symptoms might overlap with those of MS.

Patients were also selected, when possible, based on the homogeneity of their drug therapy. All participants were followed by a professional dietitian who ascertained the homogeneity of the prescribed diet. The diet was formulated based on the indications provided in the bibliography [14]. Patients enrolled for the trial followed a diet composed of major macronutrients (40% medium-chain fatty acid-poor lipids, 40% carbohydrates, and 20% protein), with a homogeneous total caloric value proportionate to individual body weight. Volunteers were given a list of foods to avoid, to reduce eating habits that could have interfered with the experiment.

### 2.2. Processing and Analysis of Blood Samples

Venous blood sampling was performed according to “WHO Guidelines on Drawing Blood, 2010”. Whole blood was immediately centrifuged at 500× *g* for 20 min after collection. Venous blood samples (5 mL) were collected at the beginning and weekly during the treatment. Serum was separated and aliquots were stored at −80 °C. The total antioxidant status (TAS), superoxide dismutase (SOD), glutathione peroxidase (GPx), and catalase (CAT) activities were determined using a previously reported method [15,16]. Several routine blood parameters were monitored (blood glucose, total cholesterol, triglycerides, total lipids, albumin, creatinine, retinol-binding protein (RBP), and C-reactive protein (CRP)). Measurement was accomplished through the CBC test using Cell Counter Sysmex XP-300 model (Sysmex, Kobe, Japan) and chemiluminescence microparticle immunoassay/Abbott biochemical method using Abbott IMX kits with Abbott IMx^®^ unit (Abbott Diagnostics, Lake Forest, IL, USA).

### 2.3. Statistical Analysis

The statistical analysis was performed using SPSS software (ver. 14.0; SPSS Inc., Chicago, IL, USA). The data were expressed as mean ± SD. The differences in anthropometric, demographic, and fatigue test scores between supplemented and placebo groups were analyzed using the Mann–Whitney U test analysis for independent measures checking the two-tailed hypothesis at significance *p*-value < 0.01. To determine whether the difference between the serum biochemical parameters means in supplemented and placebo groups were statistically significant, the confidence interval at 95% confidence level (95% CI) was calculated using *t*-statistic (*n* = 25). If the intervals between groups overlapped, the difference was considered not significant. If there was no overlap, the difference was considered significant.

## 3. Results

### 3.1. Population Characteristics

The population baseline characteristics were presented in Table 1. The mean age was 39.60 years (SD 6.06) for the total population (*N* = 50), 40.16 years (SD 5.73) for the intervention group (*n* = 25), and 39.04 years (SD 6.32) for the control placebo group (*n* = 30). The mean height was 1.69 m (SD 0.07) for the total population, 1.69 m (SD 0.08) for the intervention group, and 1.69 m (SD 0.07) for the placebo group. The mean weight was 69.04 kg (SD 9.00) for the total population, 68.64 kg (SD 7.53) for the intervention group, and 69.44 kg (SD 10.25) for the placebo group. In the total population, the mean Body Mass Index (BMI) at baseline was 24.11 kg/m^2^ (SD 3.11), 24.03 kg/m^2^ (SD 2.86) for the intervention group, and 24.20 kg/m^2^ (SD 3.34) for the placebo group. The mean duration of the MS in the total population was 16.46 years (SD 2.87), 16.04 years (SD 2.85) for the intervention group, and 16.88 years (SD 2.82) for the placebo group. Normal distribution for all the variables was assessed by visual inspection of the histograms. The *p*-value calculated for each anthropometric variable indicated that there were no differences in the general characteristics of the participants observed at baseline between the supplemented and placebo groups (Table 1).

### 3.2. Serum Biochemical Parameters

Antioxidant enzyme activities were assessed in the blood samples taken from the supplement-treated and placebo group of participants at the end of trial (Table 2). The total antioxidant status (TAS) values were significantly higher in the supplemented group (0.89 ± 0.30 U/mg protein) with respect to the placebo group (from 0.32 ± 0.20 U/mg protein), as indicated by the absence of an overlap between the 95% CI. The TAS could be indicative of oxidative stress or an increased susceptibility to oxidative damage. Each antioxidant enzymatic activity was significantly higher in the supplemented group and showed a not overlapping 95% CI: SOD (from 3.84 ± 1.80 U/mg protein in the placebo group to 6.43 ± 0.60 U/mg protein in the supplemented group), GPx (from 2.95 ± 0.56 U/mg protein to 4.95 ± 0.67 U/mg protein), and CAT (from 2.34 ± 1.37 U/mg protein to 5.24 ± 1.27 U/mg protein). These results suggested that multivitamin supplementation increases antioxidant defense after 70 days of administration of the two nutritional supplements.

The serum biochemical parameters determined after the treatment period showed a significant increase in glycemia (from 84.52 ± 10.37 in the placebo group to 94.61 ± 6.30 g/dL in the supplemented group), total cholesterol (from 182.70 ± 42.88 to 232.84 ± 52.22 mg/dL), total lipids (from 632.82 ± 130.42 to 732.86 ± 100.20 mg/dL), albumin (from 4.45 ± 0.52 to 7.45 ± 0.32 g/dL), creatinine (from 0.35 ± 0.02 to 0.83 ± 0.25), and RBP (from 2.12 ± 1.50 to 5.82 ± 1.92 mg/dL) (Table 3), while the CRP significantly decreased (from 18.45 ± 9.53 to 9.22 ± 6.42 mg/dL) (all with 95% CI not overlapped). The reduction in triglycerides, from 104.75 ± 52.72 to 112.73 ± 32.24 mg/dL, was not significant (95% CI overlapped).

### 3.3. Effects of Nutritional Supplementation on Fatigue

**Figure 1:**
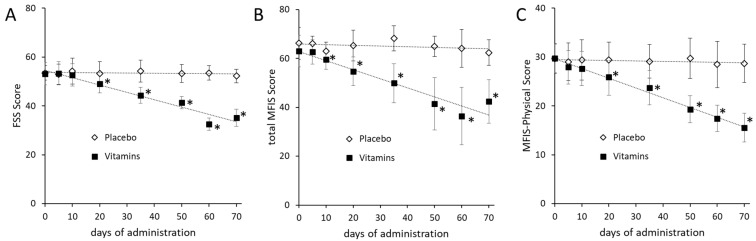
Changes in FSS (**A**), MFIS (**B**), and Physical Subscale (**C**) values in placebo and supplemented groups of MS affected subjects. The tendency curve is indicated by the dotted line. The data are expressed as mean ± SD. The *p*-values were calculated using the Mann–Whitney U test and considered significant when *p* < 0.01. Asterisks indicated perceived fatigue conditions significantly differing between supplemented and placebo groups.

Fatigue symptoms were examined using the FSS and MFIS tests to assess the effectiveness of treatment with the two dietary supplements or placebo at baseline and after 5, 10, 20, 35, 50, 60, and 70 days of treatment. The FSS test was administered to patients to assess the impact of fatigue in their daily activities (Figure 1A). After 20 days of supplements, the FSS score showed a significant decrease (*p* < 0.00001) in the group supplemented with Citozym^®^ and Ergozym^®^ (t0: mean = 53.16, SD = 4.60; t20: mean = 48.96, SD = 3.57) with respect to the pre-supplementation condition, as well as in the following days until the end of the treatment (*p* < 0.00001). No significant differences in the FSS score were observed in the placebo group. The comparison of the FSS scores pre-treatment and after 70 days of treatment with Citozym^®^ and Ergozym^®^ indicates a 34% reduction in symptomatic fatigue.

A similar result was also obtained through the MFIS test (Figure 1B). In this case, however, the effect of the vitamins’ integration on fatigue was already significantly different (*p* = 0.00932) after 10 days of treatment (t0: mean = 63.00, SD = 6.44; t10: mean = 59.52, SD = 3.94). The comparison of the MIFS scores obtained in the participants before (t0) and after 70 days of supplementation of Citozym^®^ and Ergozym^®^ (t70: mean = 42.44, SD = 8.92) showed a significant reduction (*p* < 0.00001) in symptomatic fatigue of approximately 33% in the group that received the supplements and not significant in the group that received the placebo. This reduction indicates a clear improvement in the symptom of fatigue after 70 days of supplementation with Citozym^®^ and Ergozym^®^.

As is well known, the MIFS scale is divided into the following three subscales: physical, cognitive, and psychosocial [17,18]. The MIFS physical subscale was extrapolated from the MIFS questionnaire to highlight the inherent aspect of the felt physical fatigue. Significant differences (*p* = 0.0005) of the MIFS physical subscale score were observed after 20 days of treatment with the supplements (t20: mean = 25.92; SD = 3.72) compared to the pre-treatment condition (t0: mean = 29.72, SD = 2.88) (Figure 1C). The score progressively decreased until the end of the treatment (t70: mean = 15.56, SD = 2.91), with a final reduction in the value of about 48% compared to the beginning of the treatment. No significant changes in score were observed (*p* = 0.56868) in the placebo group before (t0: mean = 29.64, SD = 3.08) and after 70 days of observation (t70: mean 28.68, SD = 3.92). These data suggest how much the symptom fatigue can influence the psychosocial and cognitive aspect of MS patients. Although, the observed increase in the lipid profile might indicate that apparently the administration of the two nutritional supplements did not affect the cognitive impairment of the patients [19].

## 4. Discussion

The combined effects of genetic predispositions such as human leukocyte antigen variants and environmental factors such as low Vitamin D levels, cigarette smoking, obesity and sun exposure on MS have been recognized recently [20,21].

Although the recent development of immunomodulatory drugs improved the treatment of non-progressive MS forms, however, some severe MS forms including secondary progressive MS do not appear to respond to treatment. Nutrition represents not only a possible factor in the pathogenesis of MS but is also considered as a complementary treatment to control the progression of the disease [22,23].

Vitamin deficiency is determinant for MS progression. The serum levels of four antioxidant vitamins—ascorbic acid, beta-carotene, retinol, and alpha-tocopherol—are significantly lower in MS patients compared to the control group [24]. In particular, vitamin D integration in MS has been well studied. A vitamin D deficiency is associated with the progression of long-term disability, while the increase in its serum level reduced brain lesions and improved timed tandem walking [25,26], high levels of vitamin D, and a diet low in saturated fat are associated with lower relapse rates in pediatric-onset multiple sclerosis [27], with a higher physical and mental QOL [28]. The role of the vitamin B complex in MS is still debated [29]. Vitamin B deficiencies result in severe myelin degeneration, leading to a loss in neuronal signal transmission, in particular vitamin B12, which is responsible for the increased homocysteine level in MS patients [30,31]. Despite the important role in cognitive function [32], the limited clinical studies do not allow us to understand whether vitamin B12 supplements could influence MS progression.

When the therapy is ineffective in MS patients, the intervention is aimed at improving the QOL of the patients and is focused on the control of symptoms, especially the particularly acute and important sense of fatigue. The data obtained in our study, which involved patients with a definitive diagnosis of relapsing–remitting MS, indicated a significant reduction in fatigue and an improvement in the QOL in individuals who received multivitamin supplementation. These clinical outcomes were accompanied by a significant increase in antioxidant capacity and a reduction in inflammatory markers.

Fatigue is the disabling symptom of excellence in MS patients with a negative impact on QOL [33] and there are no approved treatment modalities yet [34]. The pathophysiology of fatigue in MS is still poorly understood, but peripheral inflammation [35], neuroendocrine abnormalities [36,37], the glutamate level [38] and, possibly, OS can contribute to primary fatigue in MS. In our clinical study, the reduction in fatigue observed after 70 days of multivitamins supplement was accompanied by the reduction in OS, as evidenced by the increase in TAS, CAT, GPx, and SOD activity values, which suggest the reactivation of the detoxification process after the administration of Citozym^®^ and Ergozym^®^. In addition, we observed an increased serum albumin level in the multivitamin supplemented group, which is in agreement with the reduction in OS. Albumin is a powerful antioxidant with free radical scavenging activity [39], has a neuroprotective role in neurological diseases [40], and its level is reduced in MS patients [41]; therefore, its increase may have contributed to the reduction in OS in the group who received integration. Regarding the involvement of OS on the fatigue symptom in MS, very few reports are present in the literature. Sanoobar et al. reported that a dietary supplement of vitamin Q, the powerful antioxidant coenzyme Q10, reduced OS and fatigue in MS patients [42], and Kourchaki et al. evidenced that integration with ω-3 fatty acid and vitamin D reduced OS and inflammation, improving the disability score in MS patients [43], suggesting a possible correlation. However, a recent clinical trial carried out in patients with progressive MS evaluated the effect of supplementation with the antioxidant N-acetyl cysteine (NAC), without highlighting either a clinical benefit in terms of fatigue reduction nor an increase in antioxidant capacity [44], thus indicating that OS and fatigue may be unrelated events. Likewise, the benefits on fatigue we observed in MS patients supplemented with Citozym^®^ and Ergozym^®^ may not be linked to antioxidant capacity. Nonetheless, evidence of OS in the brains of patients with progressive MS [45], and the crucial role of OS in the loss of oligodendrocytes, neuronal damage, and myelin degeneration [46] suggest some possible benefits can arise, reducing the OS by Citozym^®^ and Ergozym^®^ in MS patients. In this regard, the evidence from the literature suggests the importance of vitamin C integration, since its level is decreased in MS patients and neurons are particularly sensitive to OS. Furthermore, myelin sheaths are damaged in MS and the remyelination process requires the maturation of oligodendrocytes, which is facilitated by vitamin C, which is normally present in high concentrations in the brain, particularly in the central nervous system [46].

Inflammation is involved in all stages of MS development and progression [47] and is a possible cause of fatigue in MS patients. The anti-inflammatory effect of Citozym^®^ and Ergozym^®^ has been described above [48]. The CRP, a biomarker of inflammation, can provide information on the biological state of the lesion [49]. According to the literature, the patients enrolled in our study showed elevated CRP serum levels, which gradually decreased during the administration of the multivitamin supplement, reaching significantly different values in the advanced stages of treatment. Moderate physical activity also reduces CRP levels [50,51]. An inverse relationship between the creatinine level and MS has been recently reported and serum levels of CRP and creatinine have been proposed as potential biomarkers for neurodegenerative diseases [52]. Creatinine is a biomarker directly related to muscle mass [53]. Therefore, we suggest that a reduction in inflammation and fatigue observed after treatment with the multivitamin supplement could have facilitated greater muscle activity in MS patients, and that the latter, in turn, could have reduced inflammation, thus triggering a beneficial self-sustaining mechanism leading to a further reduction in inflammation, possibly due to the greater availability of patients to movement. An improvement in physical activity also seems to emerge from the significant increase in serum creatinine observed following the administration of the multivitamin supplement, since it indicates a gain in muscle mass in the integrated group.

The role of vitamin A in MS is related to the reduction in inflammation and the increase in autoimmunity tolerance. The plasma concentration of RBP is regulated by the vitamin A status to ensure that, in vitamin A deficiency, RBP molecules are not secreted from the liver. RBP is a plasma protein belonging to α1-globulins that interacts with vitamin A to form an RBP-retinol complex that binds to plasma pre-albumin. Since serum RBP levels were found to be increased in the MS patients treated with the two nutritional supplements, it is, therefore, plausible that the administration of vitamin A, present in the two nutritional supplements, stimulated RBP production. Actually, no indication for the use of vitamin A as a treatment for MS is reported [54]. A cohort study suggested an inverse association of vitamin A levels in serum and the activity of relapsing–remitting MS [55]. These findings are, in part, derived from previous observations that the oral administration of vitamin A improves the course of experimental autoimmune encephalomyelitis [56]. In disagreement with these data, it was recently reported that ATRA therapy did not improve progressive MS and no alterations in T- and B-cell subsets were observed [57].

Despite the conflicting results reported in the literature on the use of single vitamins in the reduction in symptoms in MS patients, we can conclude that the multivitamin supplementation we tested has been shown to improve the QOL in MS patients through a significant reduction in fatigue. In fact, these patients, despite the persistence of the difficulties of the pathology, were able to perform some primary functions in their daily life that involve muscular performance, significantly improving their QOL.

## 5. Conclusions

In conclusion, the reported exploratory study suggests that the administration of some key cellular metabolism components as part of nutritional treatment may have a beneficial effect on MS-associated fatigue. However, further studies including larger cohorts of patients will be needed to confirm diet’s role in this disabling symptom in MS. Although the sample size limits the statistical significance of this study and the interpretation of the results, the comparison between the placebo and supplemented groups allows for the assessment of relevant trends and sets the basis for further investigation. The effectiveness of complementary and alternative nutritional therapies should also rely on developing feasible non-invasive markers that allow for the quantification and the implementation of disability progression scales to improve MS patients’ QOL.

## Figures and Tables

**Table 1:** Demographic and Anthropometric mean values of entire group of participants and the two single subgroups. Standard deviations indicated within the brackets. The *p*-values were calculated using the Mann–Whitney U test analysis.

Characteristic	All Sample *N* = 50	Supplemented Group *N* = 25	Placebo Group *N* = 25	*p*-Value
age (years)	39.60 (6.06)	40.16 (5.73)	39.04 (6.32)	0.4715 *
height (m)	1.69 (0.07)	1.69 (0.08)	1.69 (0.07)	0.9840 *
weight (kg)	69.04 (9.00)	68.64 (7.53)	69.44 (10.25)	0.6384 *
BMI (kg/m^2^)	24.11 (3.11)	24.03 (2.86)	24.20 (3.34)	0.8259 *
duration MS (years)	16.46 (2.87)	16.04 (2.85)	16.88 (2.82)	0.2420 *

Round brackets: SD value; * *p*-value > 0.01 was not significant.

**Table 2:** Monitoring the oxidative state of participants. Values are expressed as mean.

	Placebo Group		Supplemented Group	
TAS (U/mg protein)	0.32 (0.20)	[0.24–0.40]	0.89 (0.30)	[0.77–1.01]
SOD (U/mg protein)	3.84 (1.80)	[3.10–4.58]	6.43 (0.60)	[6.18–6.68]
GPx (U/mg protein)	2.95 (0.56)	[2.72–3.18]	4.95 (0.67)	[4.67–5.23]
CAT (U/mg protein)	2.34 (1.37)	[1.77–2.91]	5.24 (1.27)	[4.72–5.76]

Square brackets: 95% CI; Round brackets: SD value.

**Table 3:** Serum biochemical parameters. Values are expressed as mean.

	Placebo Group		Supplemented Group	
Glycemia (mg/dL)	84.52 (10.37)	[80.24–88.80]	94.61 (6.30)	[92.01–97.21]
Total Cholesterol (mg/dL)	182.70 (42.88)	[165.00–200.40]	232.84 (52.22)	[211.28–254.39]
Triglycerides (mg/dL)	104.75 (52.72)	[82.99–126.51]	112.73 (32.24)	[99.42–126.04]
Total Lipids (mg/dL)	632.82 (130.42)	[578.98–686.65]	732.86 (100.20)	[691.50–774.22]
Albumin (g/dL)	4.45 (0.52)	[4.23–4.66]	7.45 (0.32)	[7.32–7.58]
Creatinine (mg/dL)	0.35 (0.02)	[0.34–0.36]	0.83 (0.25)	[0.73–0.93]
RBP (mg/dL)	2.12 (1.50)	[1.60–2.64]	5.82 (1.92)	[5.03–6.61]
CRP (mg/L)	18.45 (9.53)	[14.52–22.38]	9.22 (6.42)	[6.57–11.87]

Square brackets: 95% CI; Round brackets: SD value.

## Data Availability

The data presented in this study are available on request from the corresponding author. The data are not publicly available due to ethical reason.
